# Exploring new potential role of DDB2 by host cell reactivation assay in human tumorigenic cells

**DOI:** 10.1186/s12885-019-6258-0

**Published:** 2019-10-29

**Authors:** Elisabetta Bassi, Paola Perucca, Isabella Guardamagna, Ennio Prosperi, Lucia A. Stivala, Ornella Cazzalini

**Affiliations:** 1 Dipartimento di Medicina Molecolare, Unità di Immunologia e Patologia generale, Università degli Studi di Pavia, Pavia, Italy; 2 Istituto di Genetica Molecolare (IGM) del CNR, Pavia, Italy

**Keywords:** DNA damage response, DNA damaged binding protein 2, Global genome nucleotide excision repair, *Xeroderma Pigmentosum* group G, RNA polymerase II

## Abstract

**Background:**

The Host Cell Reactivation assay (HCR) allows studying the DNA repair capability in different types of human cells. This assay was carried out to assess the ability in removing UV-lesions from DNA, thus verifying NER efficiency. Previously we have shown that DDB2, a protein involved in the Global Genome Repair, interacts directly with PCNA and, in human cells, the loss of this interaction affects DNA repair machinery. In addition, a mutant form unable to interact with PCNA (DDB2^PCNA-^), has shown a reduced ability to interact with a UV-damaged DNA plasmid in vitro.

**Methods:**

In this work, we have investigated whether DDB2 protein may influence the repair of a UV-damaged DNA plasmid into the cellular environment by applying the HCR method. To this end, human kidney 293 stable clones, expressing DDB2^Wt^ or DDB2^PCNA-^, were co-transfected with pmRFP-N2 and UV-irradiated pEGFP-reported plasmids. Moreover, the co-localization between DDB2 proteins and different NER factors recruited at DNA damaged sites was analysed by immunofluorescence and confocal microscopy.

**Results:**

The results have shown that DDB2^Wt^ recognize and repair the UV-induced lesions in plasmidic DNA transfected in the cells, whereas a delay in these processes were observed in the presence of DDB2^PCNA-^, as also confirmed by the different extent of co-localization of DDB2^Wt^ and some NER proteins (such as XPG), vs the DDB2 mutant form.

**Conclusion:**

The HCR confirms itself as a very helpful approach to assess in the cellular context the effect of expressing mutant vs Wt NER proteins on the DNA damage response. Loss of interaction of DDB2 and PCNA affects negatively DNA repair efficiency.

## Background

DNA damaged binding protein 2 (DDB2) plays a crucial role in DNA Damage Response (DDR) activated by UV radiation [1]. This protein is known to act as an important sensor in the Global Genome Nucleotide Excision Repair (GG-NER) by recognizing sites of UV-induced DNA lesions [2]. This function is shared with DDB1, which associates to DDB2 to form the heterodimeric UV-damaged DNA-binding protein complex (UV-DDB); this complex initiates GG-NER by recognizing photodimers (CPDs) and 6–4 photoproducts (PPs), the primary type of lesions induced by UV irradiation. The distortion of the double helix caused by the CPDs is smaller than that of 6-4PPs, and their recognition is performed by the synchronized work of UV-DDB complex and XPC protein [3]. Mutations in NER genes are linked to human genetic diseases (e.g. Xeroderma pigmentosum) as well as cancer predisposition [4–6].

The mutagenic effect of UV is efficiently neutralized by DNA repair processes involving not only GG-NER but also the transcription-coupled nucleotide excision repair (TC-NER), a sub-pathway that preferentially removes DNA lesions generated in highly transcribed DNA regions [7]. At the molecular level, both these processes are promoted and regulated by various post-translational modifications of NER factors and chromatin substrate. While GG-NER employs UV-DDB heterodimer and XPC complex to initiate the DNA repair process, TC-NER utilizes elongating RNA polymerase II (Pol II) and Cockayne syndrome B (CSB) proteins as damage sensors [8].

We have previously demonstrated that the interaction between DDB2 and PCNA is important to remove DNA lesions by NER. In fact, a mutated form of DDB2, unable to interact with PCNA (DDB2^PCNA-^), causes a delay in UV-induced NER process activation and confers proliferative and migratory advantages in HEK293 stable clone expressing DDB2^PCNA-^ [9, 10].

In addition, using gel electrophoretic motility shift assay, we showed that DDB2^Wt^ recombinant protein retains the ability to bind directly plasmidic UV-damaged DNA*,* but not the DDB2 mutated form [10]. Nevertheless, this finding does not prove that DDB2^PCNA-^ since the mutant form at the cellular level localized to DNA damage sites and interact with DDB1 [10]. To clarify this issue, we decided to apply a transfection-based assay, named Host Cell Reactivation (HCR), to investigate DNA lesions removal efficacy in the presence of DDB2^Wt^ protein or DDB2 mutated one. This method allows studying the DNA repair capability in different types of human cells [11] and may be employed as a marker for genetic instability and cancer risk [12, 13]. A subsequent adaptation to FACS technology further improved its sensitivity, compared to the previous luminometer method [14]. The HCR assay assesses repair of a transcriptionally active genes and, once applied to UV lesions, it measures the capacity of the host cells to perform NER [15].

In order to investigate whether DDB2 protein interacts with nude plasmidic UV-damaged DNA in cellular environment and whether the mutation in DDB2 interferes with DNA repair kinetic, we used two stable clones of HEK293 expressing DDB2^Wt^ or DDB2^PCNA-^. HCR assay was performed co-transfecting these cells with UV-C irradiated pEGFP-N1 and not irradiated pmRFP-N2 plasmids. To further elucidate the ability of DDB2^Wt^ and mutant form to interact with transcription machinery, co-localization to the UV-damaged sites between RNA polymerase II (Pol II), a protein sensor of DNA lesions in transcribed genes, was also considered. Finally, DDB2 recruitment and co-localization with XPG was detected to assess potential modifications in the DNA excision step kinetic.

## Methods

### Cell lines and transfection

HEK293 (Human Embryonic Kidney) cell line was purchased from the European Tissue culture Collection (ECACC) (catalogue code: 85120602). Cells were cultured in Dulbecco’s modified Eagle’s medium (DMEM, Sigma) supplemented with 10% foetal bovine serum (Life Technologies-Gibco), 2 mM l-glutamine (Life Technologies-Gibco), 100 U/ml penicillin, 100 μg/ml streptomycin in a 5% CO_2_ atmosphere.

Cell lines (50% confluent) were stably transfected with DDB2^Wt^ construct kindly provided by dr. Q. Wang [16] or the mutated form DDB2^PCNA-^ using Effectene transfection reagent (Qiagen). DDB2^PCNA-^ mutated in PIP-BOX region was produced in our laboratory, as previously described [9].

HeLa S3 cell line was purchased from European Tissue Culture Collection (ECACC, catalogue code: 87110901). HeLa cells were cultured as above, seeded on coverslips (70% confluent) and transiently transfected with DDB2 wild-type or mutated form constructs as previously described [9]. Both cell lines were periodically tested for mycoplasma contamination.

### UV plasmid preparation

pEGFP-N1 (Clontech) was irradiated in 10.5 μl of TE buffer (10 mM Tris–HCl, 1 mM EDTA, pH 8.0) at a DNA concentration of 2.85 μg/μl with 800 J/m^2^ UV-C lamp (Philips TUV-9) emitting mainly at 254 nm, as measured with a DCRX radiometer (Spectronics). Ethanol (70%) was added to DNA for the precipitation. After 15 min in freezer, DNA was centrifuged at 15500 g for 15 min at 4 °C (Allegra 21R, Beckman Coulter). The pellet was left to air dry overnight, whereas the supernatant was stored at − 20 °C. Pellet was re-suspended in 15 μl of TE buffer and the DNA was quantified by spectrophotometer (POLARstar Omega, BMG LABTECH). The supernatant was pelleted by centrifugation (Allegra 21R, Beckman Coulter) and quantified.

### Host cell reactivation assay and cytofluorimetric analysis

HEK293, stably transfected with DDB2^Wt^ or DDB2^PCNA-^ construct, were co-transfected with pmRFP-N2 (as a positive control), kindly provided by Professor Cardoso, and pEGFP-N1 or UV-pEGFP-N1 (as previously described) employing Effectene transfection reagent (Qiagen).

After 16 and 48 h, the cells were harvested from Petri dishes and centrifuged at 200 g for 3 min (Centrifuge 4236, Alc), the pellets were gently re-suspended on phosphate-buffered saline (PBS) for in vivo flow cytofluorimetry measurements (CyFlow® SL, Sysmex Partec GmbH). Only RFP positive cells were considered for the subsequent analysis in which the ratio between the mean fluorescence intensity (MFI) for the RFP and GFP protein were calculated. After normalization (MFI GFP/MFI RFP), relative expression of GFP protein was computed by comparing the normalized MFI of the UV-irradiated to the normalized MFI of unirradiated plasmid, thereby detecting the restored plasmidic DNA [14, 15].

### Immunofluorescence and confocal microscopy

HeLa cells, seeded on coverslips and transiently transfected as reported above, were locally irradiated at 100 J/m^2^ by laying on top of cells an Isopore polycarbonate filters (Millipore) with 3 μm pores. At the end of 5, 10, 30 and 60 min (recovery time for XPG) or 30 and 60 min (recovery time for Pol II), the cells were washed twice in cold PBS, lysed with 0.5% Triton X-100 (Sigma-Aldrich) in cold PBS for 30 min at 4 °C in agitation, fixed with freshly made 2% paraformaldehyde and preserved in Ethanol (70%) at − 20 °C for permeabilization.

Next, the samples were washed twice with cold PBS and blocked in PBST buffer (PBS, 0.2% Tween 20) containing 1% bovine serum albumin (BSA) with gentle shaking, and then incubated for 1 h with specific antibodies: mouse monoclonal anti-RNA polymerase II (anti-Pol II, 1:100, Covance, RRID:AB_10013665), rabbit polyclonal anti-XPE/DDB2 (1:100, Novus Biologicals; NBP2–38854) and rabbit polyclonal anti-XPG (1:200, RRID: AB_1080609), all diluted in PBST buffer/BSA. After three washing, each reaction was followed by incubation for 30 min with anti-mouse Alexa Fluor 594 (1:200, RRID: AB_141607) or anti-rabbit Alexa Fluor 488 (1:200, Molecular Probes, RRID: AB_141708). After immunoreactions and washing, the samples were incubated with Hoechst 33258 dye (0.5 μg/ml) for 10 min at room temperature with mild agitation and then washed in PBS. Slides were mounted in Mowiol (Calbiochem) containing 0.25% 1,4-diazabicyclo-octane (Aldrich) as antifading agent. Images of fixed cells were taken with a Nikon Eclipse E400 fluorescence microscope equipped with a Canon Power Shot A590 IS digital camera. Fluorescence signals were acquired with a TCS SP5 II Leica confocal microscope, at 0.3 μm intervals. Image analysis was performed using the LAS AF software.

## Results

### DNA damage response is delayed in the presence of DDB2 mutated protein

**Fig. 1:**
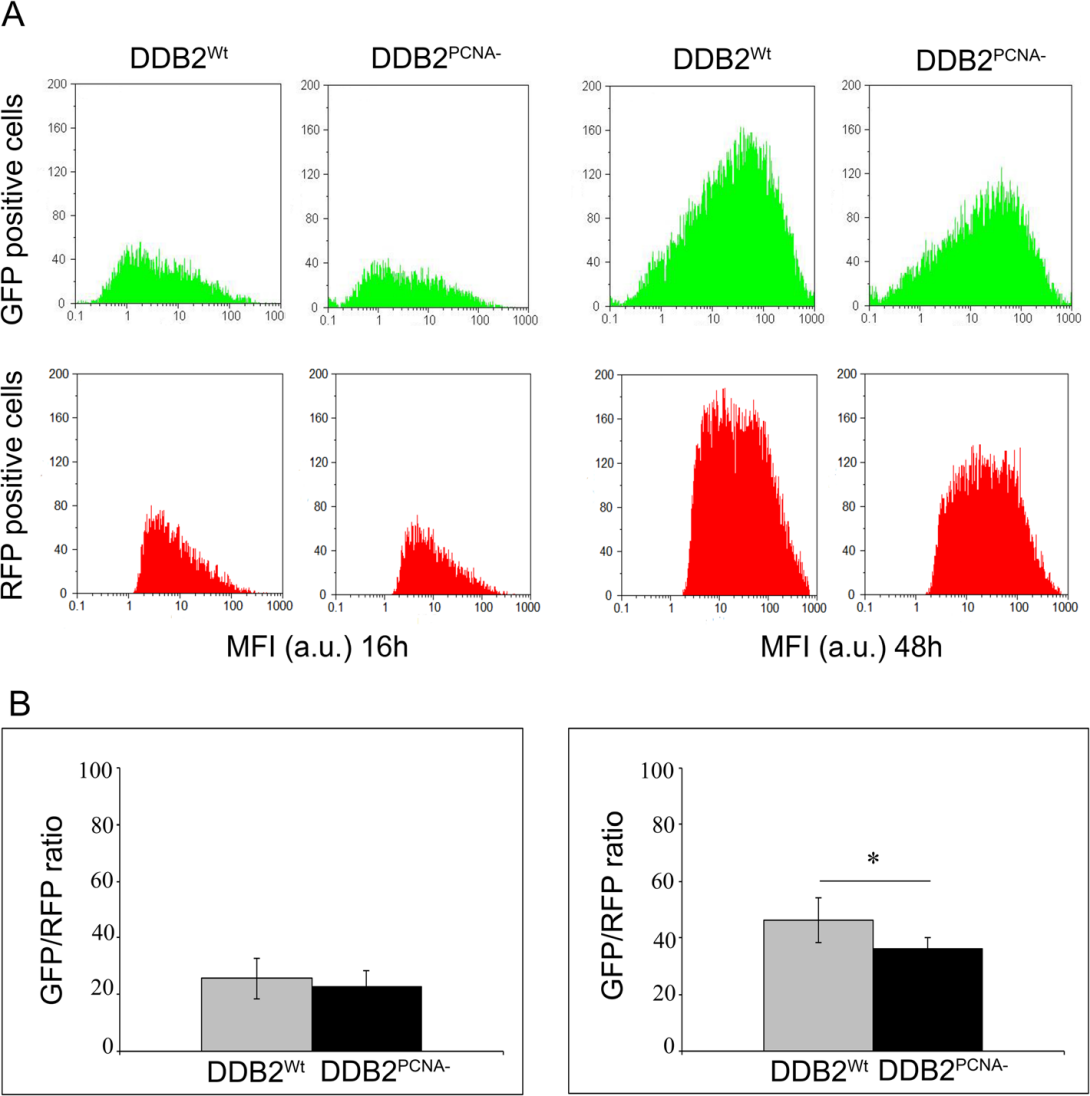
NER process is impaired when DDB2-PCNA interaction is lost. HEK293 stable clones expressing DDB2 wild-type (DDB2^Wt^) or mutated protein (DDB2^PCNA-^) were transiently transfected with pEFGP-N1 UV-irradiated plasmid and pmRFP-N2 control plasmid. At 16 and 48 h after transfection, the cells were collected and analysed using in vivo cytofluorimetric protocol (see Materials and Methods section). In **a** GFP and RFP positive cells are reported and the M.F.I. is shown. **b** Statistical analysis of GFP/RFP ratio from three independent experiments is reported. * *p* < 0.05

To evaluate the UV-induced DNA damage response, we performed experiments using irradiated or not irradiated pEGFP-N1 plasmid co-transfected with pmRFP-N2 construct in HEK293 stable clones expressing DDB2^Wt^ or DDB2^PCNA-^ protein. Flow cytometry analysis of GFP and RFP expression, performed at 16 and 48 h after plasmidic DNA-damaged transfection, highlights the production of the green fluorescent protein, indicating the ability of these cells to repair DNA lesions in irradiated pEGFP-N1 plasmid (Fig. 1). In the panel A, monoparametric analysis of the green (GFP positive cells) and red (RFP positive cells) shows no significant differences in the two cell clones at 16 h after transfection. At this time, the presence of DDB2 mutated protein does not influence the repair ability since it produced similar results as wild-type protein. In contrast, the analysis performed 48 h after transfection highlights a significant reduction of DNA damage repair capability in the presence of the mutated protein (Fig. 1b). Considering the ratio of GFP/RFP fluorescence, the GFP protein synthesis is more efficient in the presence of DDB2^Wt^; instead, the loss of DDB2-PCNA interaction determines a reduction of reported gene reactivation after UV irradiation.

### DDB2 and RNA polymerase II co-localization is prevented without PCNA involvement

**Fig. 2:**
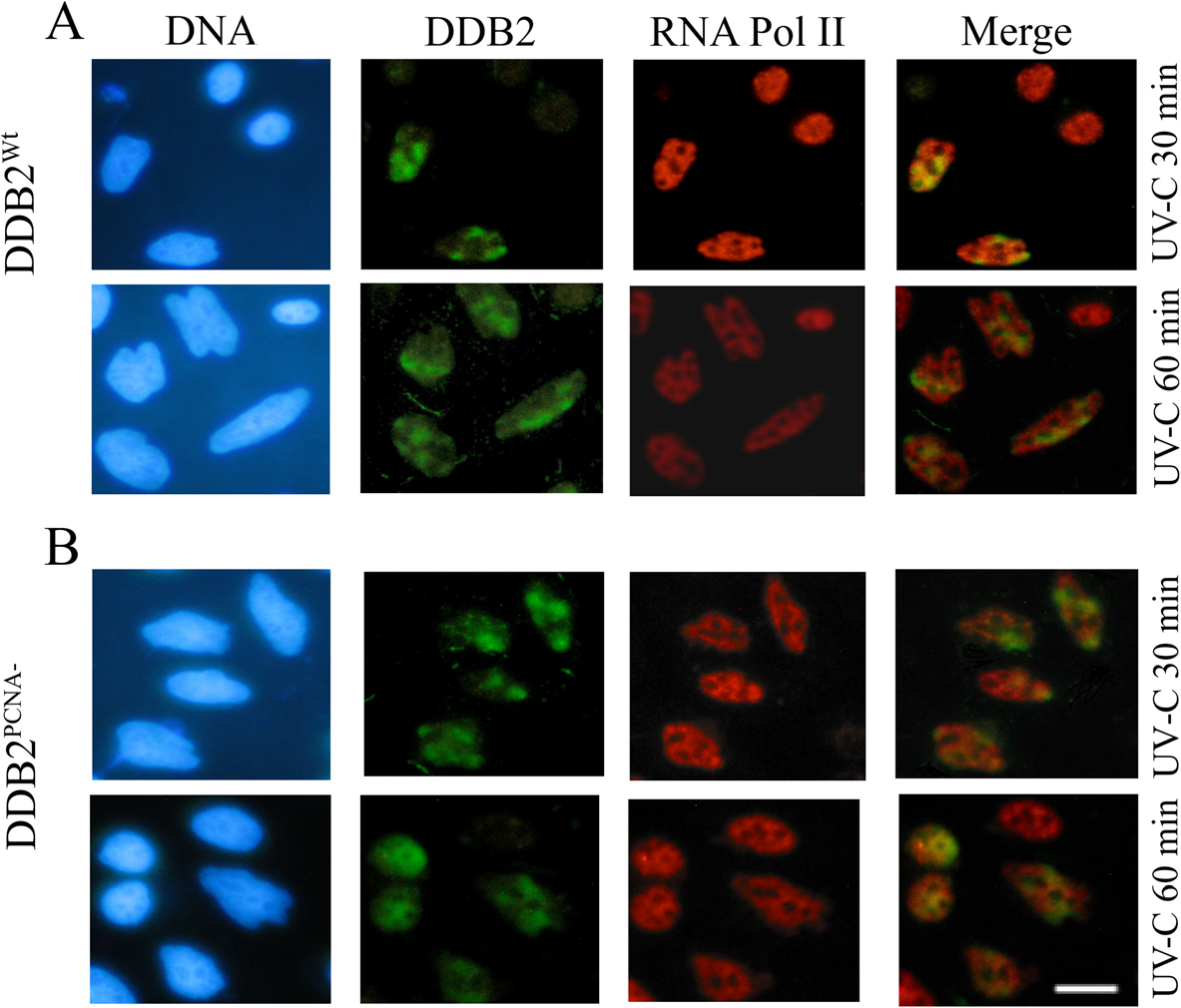
DDB2^PCNA-^ and Polymerase II proteins partially co-localize in irradiated cells. Immunofluorescence analysis of HeLa cells transiently transfected with constructs expressing DDB2^Wt^ or DDB2^PCNA-^ protein, as indicated. Cells were UV-C local irradiated and analysed 30 min and 1 h later. In **a** representative images obtained from cells expressing DDB2^Wt^ protein. In **b** results from HeLa transfected with pc-DNA3.1-DDB2^PCNA-^ construct are shown. Scale bar = 20 μm. Data are at least from three independent experiments

HeLa cells transiently transfected with pc-DNA3.1-DDB2^Wt^ or pc-DNA3.1-DDB2^PCNA-^ constructs were incubated with anti-DDB2 and anti-RNA Pol II antibodies for 30 min and 1 h after UV-C local irradiation. The immunofluorescence analysis shows that DDB2^Wt^ and Pol II were already recruited at DNA damaged sites at 30 min after DNA damage, and their co-localization were still detectable at 1 h after UV irradiation, even if the signal appears to be reduced (Fig. 2a). In the presence of DDB2 mutated protein, the cells did not show well-defined spots of co-localization at both 30 and 60 min (Fig. 2b).

**Fig. 3:**
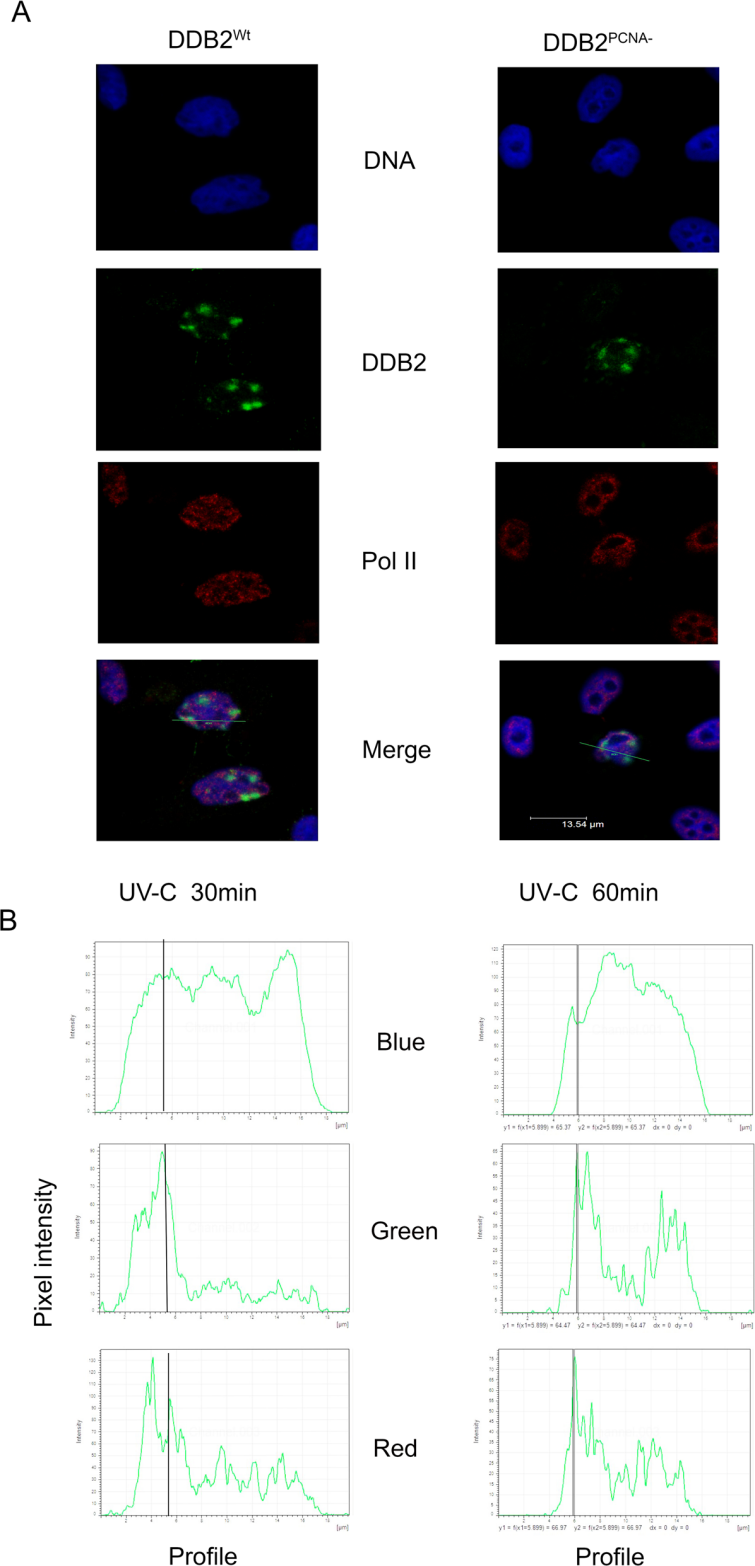
The loss of DDB2-PCNA interaction determines defects in NER pathway. HeLa cells transiently transfected with pc-DNA3.1-DDB2^Wt^ or pc-DNA3.1-DDB2^PCNA-^ constructs and UV-C local irradiated were analysed 30 min and 1 h after damage. In **a** representative co-localization analysis between DDB2 and Polymerase II proteins after UV-induced damages as obtained by confocal microscopy. The co-localization analyses are reported in panel (**b**). Data are at least from three independent experiments

To better evaluate the recruitment kinetics at DNA damaged sites of the above proteins, confocal analysis was performed as shown in Fig. 3. The co-localization between DDB2^Wt^ and Pol II occurs mainly at 30 min after UV irradiation (Fig. 3a); at this time, 52% of cells were positive for colocalization. On the contrary, in the presence of DDB2^PCNA-^ protein, only 1 h after damage, the pixel intensity profile showed a not complete co-localization. In fact, the green and red signals were partially overlapped (Fig. 3b). This finding confirms a delay in this co-presence at DNA damage foci.

### DDB2-PCNA interaction facilitates the appropriate maintenance of the late NER-phase

To evaluate the potential influence of DDB2-PCNA interaction on the late NER steps, we investigate the interaction between DDB2 and XPG, a protein involved in the incision phase of NER process. HeLa cells transiently expressing DDB2^Wt^ or DDB2^PCNA-^ protein were local irradiated and analysed by fluorescence and confocal microscopies at different recovery times.

**Fig. 4:**
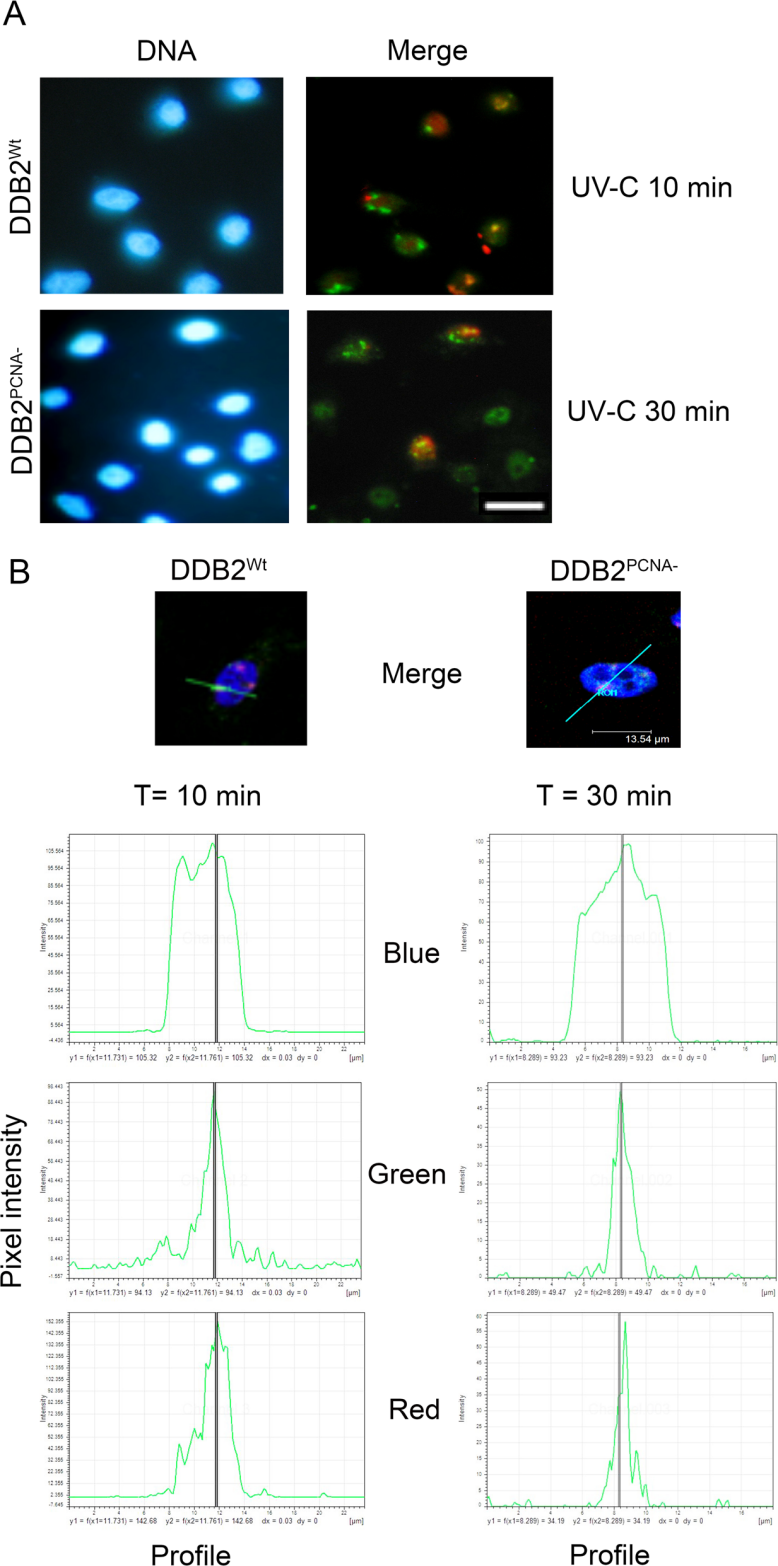
DDB2-PCNA binding influences the late phase of repair process. Representative analysis obtained from transfected and irradiated HeLa cells using immunofluorescence microscope (**a**). Scale bar = 20 μm. In **b** confocal images and analysis are reported. The peaks exemplify the co-localization studies between DDB2 and XPG proteins. Data are at least from three independent experiments

Figure 4a shows representative images of the immunofluorescence analysis. The time course after irradiation indicate that DDB2^Wt^ protein is recruited at DNA damaged sites together with the endonuclease XPG. Confocal microscopy confirmed a better co-localization between DDB2^Wt^ and XPG proteins at 10 min after UV irradiation (Fig. 4b), whereas the recruitment at the damage sites appears postponed at 30 min with regards to XPG and DDB2^PCNA-^. Furthermore, in the last case, the confocal analysis indicated that the two proteins recruited at DNA damaged sites are very closed but not completely overlapped since the profile of the peak intensity reveal that the better fluorescent signals are not superimposable. These data demonstrate that the loss of DDB2-PCNA interaction influences the late phase of NER process.

## Discussion

NER process is a highly versatile and complex system removing different types of DNA lesions [17]. UV-induced damages trigger NER process using both sub-pathways TC-NER and GG-NER. The first one is fast and efficient in removing lesions from transcribed regions determining a block of transcription [18].

The role of DDB2 in GG-NER is widely described and this protein is crucial to recognize and remove DNA UV-lesions [19, 20]. We have previously demonstrated that DDB2-PCNA interaction is i) required for DDB2 degradation [9], ii) likely involved in cell cycle progression [21], iii) able to affects DNA repair and iv) implicated in conferring proliferation and migration advantages [10]. In addition, using UV-damaged plasmidic DNA, DDB2^PCNA-^ recombinant protein showed both defective lesion recognition and DNA binding [10].

In this work we applied HRC assay to evaluate plasmidic DNA repair capacity of DDB2 protein and its mutated form. In the past, several approaches based on transfected damaged-DNA have been used to this end. After the initial demonstration on HCR efficiency for studying NER process activation in whole cells [11] or fractionated cell extracts transfected with UV-damaged plasmid DNA [11], different attempts to improve HCR assay have been further developed. Among them, a fluorescent method for detecting cellular ability to incise the damaged strand by NER mechanism [22], as well as a plasmid-type fluorescent probe [23] were proposed.

Based on our results, the re-activated expression of GFP protein in the stable clone producing DDB2^Wt^ demonstrated that DNA lesions are removed from transfected irradiated plasmidic DNA and, therefore, the transcription process is restored. It is known from the literature, that HCR assay, when performed after UV damage, measures the ability of the host cells to complete NER [15]. Our results demonstrated that this capability is influenced by DDB2-PCNA interaction; in fact, the cells expressing DDB2^PCNA-^ protein showed a significant reduction of GFP expression, as shown by the low GFP protein level measured by flow cytometry. By this experimental approach we demonstrated that both DDB2^Wt^ and DDB2^PCNA-^ proteins may intervene on nude UV-damaged plasmidic DNA inserted in human cells. Importantly, the DDB2^PCNA-^ mutant protein causes a delayed repair, confirming our previously published data obtained in an in vitro model [10]. In addition, our data support that the HCR method can be an efficient tool for investigating the role of NER mutant proteins in DNA repair. One advantage of this technical approach is that only the transfected DNA is damaged, while host cells are not irradiated and, therefore, they own intact cellular synthesis machinery and biochemical processes.

Interestingly, the co-localization between DDB2^Wt^ and RNA Pol II protein, as highlighted by confocal analysis at 30 min after UV irradiation, allows us to confirm the co-presence of these proteins at DNA damaged sites. This finding suggests a putative cooperation in DNA repair processes between TC-NER and GG-NER. Cooperation between other repair pathways have already been reported, as well as functional links between apparently separate signalling pathways converging toward a single global DNA damage response [24, 25]. In the presence of DDB2 mutated protein this cooperation is slower and its co-localization with RNA Pol II at DNA damaged sites appears incomplete even one hour after irradiation, thus suggesting a delay in the repair process.

To verify whether this different DNA damage response occurs also in the next phase of the NER and, in particular, in the excision of DNA lesions, DDB2-XPG co-localization was also considered. Although early report indicated that DDB2 is not required for XPG recruitment [26], this does not mean that co-localization may occur thereafter, as suggested by our results with DDB2^Wt^ and further supported by the evidence that loss of DDB2-PCNA interaction determines a delay on the XPG recruitment on DNA lesions. Since XPG-mediated excision of DNA containing the lesions is fundamental for the fast DNA re-synthesis to correct the errors [27], our results suggest that DDB2 may influence not only the recognition, but also the next step of the NER, confirming the results observed in delayed GG-NER process [9, 10].

## Conclusions

In conclusion, this work reports two new findings. First, the HCR data allowed highlighting the importance of the DDB2-PCNA interaction to complete correctly NER process. The second result is that HCR approach is useful to study how mutations in NER proteins may influence genome stability.

## Data Availability

The datasets used and/or analysed during the current study are available from the corresponding authors on reasonable request.

## Abbreviations

DDB2: DNA damaged binding protein 2
DDR: DNA Damage Response;
GG-NER: Global Genome Nucleotide Excision Repair
HCR: Host Cell Reactivation assay
HEK: Human Embryonic Kidney
MFI: Mean Fluorescence Intensity
PCNA: Proliferating Cell Nuclear Antigen
Pol II: RNA Polymerase II
TC-NER: Transcriptional Coupled Nucleotide Excision Repair
XPG: *Xeroderma Pigmentosum* group G

## References

[1] Wang QE, Zhu Q, Wani G, Chen J, Wani AA (2004). UV radiation-induced XPC translocation within chromatin is mediated by damaged-DNA binding protein, DDB2. Carcinogenesis.

[2] Aboussekhra A, Wood RD (1995). Detection of nucleotide excision repair incisions in human fibroblasts by immunostaining for PCNA. Exp Cell Res.

[3] Wakasugi M, Kawashima A, Morioka H, Linn S, Sancar A, Mori T, Nikaido O, Matsunaga T (2002). DDB accumulates at DNA damage sites immediately after UV irradiation and directly stimulates nucleotide excision repair. J Biol Chem.

[4] Cleaver JE, Lam ET, Revet I (2009). Disorders of nucleotide excision repair: the genetic and molecular basis of heterogeneity. Nat Rev Genet.

[5] Basu Ashis (2018). DNA Damage, Mutagenesis and Cancer. International Journal of Molecular Sciences.

[6] Broustas CG, Lieberman HB (2014). DNA damage response genes and the development of cancer metastasis. Radiat Res.

[7] Lagerwerf S, Vrouwe MG, Overmeer RM, Fousteri MI, Mullenders LH (2011). DNA damage response and transcription. DNA Repair (Amst).

[8] Mullenders LHF (2018). Solar UV damage to cellular DNA: from mechanisms to biological effects. Photochem Photobiol Sci.

[9] Cazzalini O, Perucca P, Mocchi R, Sommatis S, Prosperi E, Stivala LA (2014). DDB2 association with PCNA is required for its degradation after UV-induced DNA damage. Cell Cycle.

[10] Perucca P, Mocchi R, Guardamagna I, Bassi E, Sommatis S, Nardo T, Prosperi E, Stivala LA, Cazzalini O (2018). A damaged DNA binding protein 2 mutation disrupting interaction with proliferating-cell nuclear antigen affects DNA repair and confers proliferation advantage. Biochim Biophys Acta Mol Cell Res.

[11] Roguev A, Russev G (2000). Two-wavelength fluorescence assay for DNA repair. Anal Biochem.

[12] Wei Q, Spitz MR (1997). The role of DNA repair capacity in susceptibility to lung cancer: a review. Cancer Metastasis Rev.

[13] Ankathil R, Jyothish B, Madhavan J, Nair MK (1999). Deficient DNA repair capacity: a predisposing factor and high risk predictive marker in familial colorectal cancer. J Exp Clin Cancer Res.

[14] Burger K, Matt K, Keiser N, Gebhard D, Bergemann J (2010). A modified fluorimetric host cell reactivation assay to determine the repair capacity of primary keratinocytes, melanocytes and fibroblasts. BMC Biotechnol.

[15] Johnson JM, Latimer JJ (2005). Analysis of DNA repair using transfection-based host cell reactivation. Methods Mol Biol.

[16] Barakat BM, Wang QE, Han C, Milum K, Yin DT, Zhao Q, Wani G, Arafa E-S, El-Mahdy MA, Wani AA (2010). Overexpression of DDB2 enhances the sensitivity of human ovarian cancer cells to cisplatin by augmenting cellular apoptosis. Int J Cancer.

[17] Kusakabe M, Onishi Y, Tada H, Kurihara F, Kusao K, Furukawa M, Iwai S, Yokoi M, Sakai W, Sugasawa K (2019). Mechanism and regulation of DNA damage recognition in nucleotide excision repair. Genes Environ.

[18] Wang W, Xu J, Chong J, Wang D (2018). Structural basis of DNA lesion recognition for eukaryotic transcription-coupled nucleotide excision repair. DNA Repair.

[19] Stoyanova T, Roy N, Kopanja D, Bagchi S, Raychaudhuri P (2009). DDB2 decides cell fate following DNA damage. Proc Natl Acad Sci U S A.

[20] Stoyanova T, Roy N, Kopanja D, Raychaudhuri P, Bagchi S (2009). DDB2 (damaged-DNA binding protein 2) in nucleotide excision repair and DNA damage response. Cell Cycle.

[21] Perucca P, Sommatis S, Mocchi R, Prosperi E, Stivala LA, Cazzalini O (2015). A DDB2 mutant protein unable to interact with PCNA promotes cell cycle progression of human transformed embryonic kidney cells. Cell Cycle.

[22] Toga T, Kuraoka I, Watanabe S, Nakano E, Takeuchi S, Nishigori C, Sugasawa K, Iwai S (2014). Fluorescence detection of cellular nucleotide excision repair of damaged DNA. Sci Rep.

[23] Tawarahara H, Kuraoka I, Iwai S (2017). Facile preparation of a fluorescent probe to detect the cellular ability of nucleotide excision repair. Anal Biochem.

[24] Simonelli V, Leuzzi G, Basile G, D'Errico M, Fortini P, Franchitto A, Viti V, Brown AR, Parlanti E, Pascucci B (2017). Crosstalk between mismatch repair and base excision repair in human gastric cancer. Oncotarget.

[25] Fortini P, Dogliotti E (2007). Base damage and single-strand break repair: mechanisms and functional significance of short- and long-patch repair subpathways. DNA Repair (Amst).

[26] Zotter A, Luijsterburg MS, Warmerdam DO, Ibrahim S, Nigg A, van Cappellen WA, Hoeijmakers JH, van Driel R, Vermeulen W, Houtsmuller AB (2006). Recruitment of the nucleotide excision repair endonuclease XPG to sites of UV-induced dna damage depends on functional TFIIH. Mol Cell Biol.

[27] Riedl T, Hanaoka F, Egly JM (2003). The comings and goings of nucleotide excision repair factors on damaged DNA. EMBO J.

